# Association of Breakfast Quality and Energy Density with Cardiometabolic Risk Factors in Overweight/Obese Children: Role of Physical Activity

**DOI:** 10.3390/nu10081066

**Published:** 2018-08-10

**Authors:** Lide Arenaza, Victoria Muñoz-Hernández, María Medrano, Maddi Oses, Maria Amasene, Elisa Merchán-Ramírez, Cristina Cadenas-Sanchez, Francisco B. Ortega, Jonatan R. Ruiz, Idoia Labayen

**Affiliations:** 1 Institute for Innovation & Sustainable Development in Food Chain (IS-FOOD), Public University of Navarra, 31006 Pamplona, Spain; maria.medrano@unavarra.es (M.M.); maddi.oses@unavarra.es (M.O.); idoia.labayen@unavarra.es (I.L.); 2 PROmoting FITness and Health through physical activity research group (PROFITH), Department of Physical and Sports Education, Faculty of Sport Sciences, University of Granada, 18071 Granada, Spain; mariavmuher@gmail.com (V.M.-H.); elymerchan@hotmail.com (E.M.-R.); cristina.cadenas.sanchez@gmail.com (C.C.-S.); ortegaf@ugr.es (F.B.O.); ruizj@ugr.es (J.R.R.); 3 Department of Pharmacy and Food Sciences, University of the Basque Country of the Basque Country, UPV/EHU, 01006 Vitoria-Gasteiz, Spain; mariamasene10@gmail.com

**Keywords:** breakfast quality, breakfast energy density, skipping breakfast, cardiometabolic health, childhood obesity, uric acid, HOMA, cholesterol, blood pressure, physical activity

## Abstract

There is a general belief that having breakfast is an important healthy lifestyle factor; however, there is scarce evidence on the influence of breakfast quality and energy density on cardiometabolic risk in children, as well as on the role of physical activity in this association. The aims of this paper were (i) to examine the associations of breakfast quality and energy density from both solids and beverages with cardiometabolic risk factors, and (ii) to explore whether physical activity levels may attenuate these relationships in children with overweight/obesity from two projects carried out in the north and south of Spain. Breakfast consumption, breakfast quality index (BQI) score, BEDs/BEDb (24 h-recalls and the KIDMED questionnaire), and physical activity (PA; accelerometry) were assessed, in 203 children aged 8–12 years who were overweight or obese. We measured body composition (Dual X-ray Absorptiometry), uric acid, blood pressure, lipid profile, gamma-glutamyl-transferase (GGT), glucose, and insulin, and calculated the HOMA and metabolic syndrome *z*-score. The BQI score was inversely associated with serum uric acid independently of a set of relevant confounders (β = −0.172, *p* = 0.028), but the relationship was attenuated after further controlling for total PA (*p* < 0.07). BEDs was positively associated with total and HDL cholesterol, and systolic blood pressure regardless of confounders (all *p* < 0.05), while BEDb was positively associated with HOMA in either active/inactive children (all *p* < 0.03). In conclusion, higher breakfast quality and lower breakfast energy density should be promoted in overweight/obesity children to improve their cardiometabolic health.

## 1. Introduction

Breakfast has traditionally been considered as being not only the first, but also the most important meal of the day. Skipping breakfast is associated with excess adiposity during childhood [1,2,3] and therefore, breakfast consumption has been suggested as a childhood obesity-preventing strategy [4]. Indeed, it has been reported that children are hungrier and consume more food before lunch when skipping breakfast, while it is unclear when skipping other meals [5]. The overall diet quality of children who consume breakfast is healthier compared to those who skip it [6]. Skipping breakfast prevalence increases with age [7], and it is associated with increased abdominal [8] and visceral adiposity [9], metabolic syndrome [10], and lower levels of physical activity [11].

Currently, on account of the wide variety of food available, ultra-processed foods are common in childrens’ diets. The breakfast of Spanish children is predominantly composed of sugary and fat-rich products such as biscuits, pastries, breakfast cereals, artificial juices, and milkshakes [12]. In this line, a previous study reported that breakfast frequency and quality may affect glycaemia and appetite in both children and adults [13]. Thus, Monteagudo et al. proposed a Breakfast Quality Index (BQI), an itemized tool based on the well-known Mediterranean dietary pattern, to assess breakfast quality in children and adolescents [14]. Recently, energy density has been used as dietary parameter to evaluate the associations between diet and adiposity [15]. A previous study suggested to analyze energy density from solids (BEDs) and beverages (BEDb) separately, since excluding caloric beverages from BEDs calculation seems to be a useful method to avoid misinterpretation of true exposure to a high energy dense diet [16]. Thereby, diet quality and energy density are important dietary factors when analyzing the adequacy of breakfast as well as of the other meals such as lunch or dinner.

Physical activity is associated with a lower risk of cardiovascular disease [17], and specially, with insulin resistance [18]. Moreover, previous studies observed that high physical activity levels may reduce the detrimental effect of unhealthy dietary patterns in children and adolescents [19]. Previous studies concluded that youth who seldom ate breakfast tended to be more sedentary [20,21]. According to a Swedish study, children aged 10 years consuming a breakfast which provided over 20% of their estimated daily energy requirements, performed significantly better in before-lunch physical endurance tests compared to children who consumed a breakfast providing with only 10% of their energy requirements [22]. However, there is no strong evidence on whether having a low energy intake breakfast time is detrimental to childrens’ physical activity levels [23]. 

Therefore, given that both the quality and the energy density seem to be important dimensions of the first meal of the day, to be considered for their potential relationship between obesity and related metabolic disorders, and that physical activity may attenuate the detrimental effect of unhealthy breakfast habits on cardiometabolic health, the aims of the current study were: (i) to examine the associations of breakfast quality and energy density from both solids and beverages with cardiometabolic risk factors, and (ii) to explore whether physical activity levels may attenuate these relationships in children who were overweight or obese from two projects carried out in the north and south of Spain.

## 2. Material and Methods

### 2.1.Study Design and Participants 

The current cross-sectional pooled analysis includes baseline data from the EFIGRO (ClinicalTrials.gov ID: NCT02258126) [24] and the ActiveBrains (ClinicalTrials.gov ID: NCT02295072) [25] randomized controlled trials. Both studies involve participants with the same characteristics and were conducted between 2014 and 2017. The EFIGRO study was carried out in Vitoria-Gasteiz (North of Spain), with the aim of examining the additional effect of supervised exercise training on hepatic fat content in children with overweight/obesity participating in a family-based healthy lifestyle educational intervention. The ActiveBrains project was conducted in the southern Spanish city of Granada with the main objective to investigate the effect of a supervised exercise training program on brain, cognition, and academic achievement as well as physical and mental health in children with overweight/obesity. The study protocols were approved by the Ethic Committee of Clinical Investigation of Euskadi (PI2014045) and the Review Committee for Research Involving Human Subjects at University of Granada (Reference: 848, February 2014), respectively. Parents or legal guardians whose child was participating in the study signed an informed written consent before being enrolled in the study. 

For the current cross-sectional study we used the baseline data of a total of 203 children (47.3% girls) with overweight/obesity, aged 8–12 years and with dietary data available from two nonconsecutive 24 h-recalls were included in the analysis.

### 2.2. Anthropometry, Cardiometabolic Risk Assessment and Socio-Demographic Data 

Height (SECA 220, Hamburg, Germany) and body weight (SECA 760, Hamburg, Germany) were measured barefoot following the standard protocols. Body mass index (BMI) was calculated as body weight divided by height squared (kg/m^2^). According to World Obesity Federation criteria, children were classified as being overweight or obese [26]. Waist circumference was measured by a nonelastic tape (SECA 201, Hamburg, Germany) after a normal breathing in standing position, at the middle point between the anterior iliac crest and lower border of the rib. Total and abdominal fat were assessed by dual energy X-ray absorptiometry (DXA; HOLOGIC, QDR 4500W, and HOLOGIC, Discoveri Wi for EFIGRO and ActiveBrains, respectively) and FMI was calculated as fat mass divided by height squared (kg/m^2^). Abdominal adiposity was measured by determining three abdominal sections as described elsewhere [27]. 

Systolic and diastolic blood pressure (OMRON^®^ M6) were measured twice following recommendations for children [28]. Puberty stage was determined by direct examination by a pediatrician according to Tanner and Whitehouse [29]. Biochemical parameters such as serum lipid profile, gamma glutamyl transferase (GGT), glucose, insulin, and uric acid were obtained from fasting blood samples as has been reported elsewhere [24,25]. Insulin resistance was determined by computing homeostatic model assessment (HOMA) as follows: (insulin (µU/mL) × glucose (mmol/dL))/22.5) [30]. Maternal educational level was recorded and categorized as high (university or higher education) or low (primary, secondary, or high school). 

Children were categorized as having or not metabolic syndrome according to the IDF criteria [31,32]. According to this definition, children were considered as having MetS if they presented three or more metabolic risk factors (central obesity; elevated systolic (≥130 mmHg) or diastolic (≥85 mmHg) blood pressure; elevated triglycerides (≥150 mg/dL) or low HDL cholesterol (<40 mg/dL); and impaired fasting blood glucose (≥100 mg/dL)). We calculated the metabolic syndrome *z*-score based on sex- and age-specific *z*-scores using data from a sample of Spanish children with a similar age range and with all BMI categories [33]. MetS *z*-score was computed by using the sum of the sex- and age-specific *z*-scores for the most common variables included in the metabolic syndrome definition (triglycerides, HDL-cholesterol, glucose, and systolic and diastolic blood pressure) [34].

### 2.3. Breakfast Intake, Quality and Energy Density Assessment

Two nonconsecutive 24 h-recalls of weekdays were recorded during a time span of a week by trained nutritionists, and thereafter, the mean of energy intake as well as food intake of both days was calculated. Children reported their detailed intake from the previous day in an interview with trained nutritionists and with the collaboration of their parents. Parents were not aware of the dietary assessment previous to the interview in order to avoid reporting bias. Pictures of food servings were used to estimate servings and food sizes [35]. Nutritional composition data was obtained by the Easydiet computer software (© Biocentury, S.L.U. 2016), which is supported by the Spanish Association of Dietetics and Nutritionists. Those participants with a single questionnaire or with noncompleted data on dietary intake were excluded from the analyses (*N* = 22). 

Skipping breakfast information was obtained (i) extracting an item from the KIDMED questionnaire [36], and (ii) based on meals recorded in the 24 h-recalls. In addition, the quality of the breakfast was evaluated based on the modified tool “Breakfast Quality Index (BQI)” proposed by Monteagudo C et al. [14], which was slightly modified. The index used in our study is composed of 10 items related to breakfast consumption and is based on whether or not children meet the criteria of each component. The criteria to score one point in each item are the following: to consume (i) cereals and derivates, (ii) fruits and/or vegetables, (iii) dairy products, (iv) to provide <5% of total daily energy from food rich in simple sugars from breakfast, (v) to consume olive oil, (vi) monounsaturated to saturated fatty acids ratio (MUFA/SFA) ≥2:1, (vii) to provide 20–25% of energy from breakfast in relation to total daily energy intake, (viii) to consume cereals, fruits, and dairy products at the same meal, (ix) to consume ≥200–300 mg of calcium, and (x) not to consume butter or margarine. Thereby, 1 point was scored when children met the criterion in each item, while a value of 0 was given when this was not achieved. The BQI score ranged from 0 (poorest breakfast quality) to 10 points (optimal breakfast quality). Detailed information of the BQI score is available in Supplemental Table S1.

Breakfast energy density from both solids and beverages was calculated by dividing the energy consumed (kcal) by the amount (mass in grams) of food or drinks consumed, respectively (kcal/g). Breakfast energy density from solids (BEDs) includes all solid food excluding all energy-containing and non-energy-containing beverages, whereas breakfast energy density from beverages (BEDb) included all drinkable energy-containing beverages such as soft drinks, milk, smoothies, shakes, or juices. 

### 2.4. Physical Activity Assessment

Accelerometers were used to objectively measure total physical activity (wActisleep-BT and wGT3X-BT, ActiGraph, Pensacola, FL, USA). Children had to wear the accelerometer on the nondominant wrist during a seven consecutive days (24 h) except during water-activities. In order to quantify the acceleration related to the movement registered and expressed in milligravity (mg), Euclidean Norm Minus One (ENMO) was analyzed using the R software (GGIR Packae, v. 1.5-12, https://cran.r-project.org/web/packages/GGIR/). Moderate to vigorous physical activity (MVPA) was estimated by establishing age-specific cut-offs for ENMO [37] as recommended elsewhere [38]. Children were considered as active when meeting the criteria of reaching a minimum of 60 min of MVPA per day and as inactive when MVPA <60 min/day [39].

### 2.5. Statistical Analysis

The distribution of the variables was analyzed by exploring the skewness and kurtosis, and as triglycerides, GGT, insulin, and HOMA that did not have a normal distribution, all were logarithmically transformed. As the DXA used in both of the studies to assess total and abdominal adiposity was similar but not exactly the same model, *z*-score of FMI and abdominal adiposity according to the study center was calculated in order to avoid any possible systematic error. To examine the association of breakfast quality with cardiometabolic risk factors linear regression analyses were performed considering breakfast quality as independent variable and cardiometabolic risk factors as dependent variables. For this purpose, three models were performed; sex, age, center (Vitoria or Granada), maternal educational level, and energy intake were used in Model 1; Model 2 was additionally adjusted for FMI, and in Model 3, the analysis was further adjusted for total physical activity. This last was included in the third model to explore if physical activity plays any role in the association between breakfast dimensions and cardiometabolic risk. Linear regression analyses were also performed to examine the association of energy density from solids and beverages (independent variables) with cardiometabolic risk factors and MetS *z*-score (dependent variables). The same models were used for both objectives. As sensitivity analyses, all the regression models were repeated including the puberty stage instead of age as confounder. Sex interaction was also examined and no significant differences were found, thus, the results are presented with boys and girls together. Statistical analyses were carried out with the statistical software SPSS version 20.0 (SPSS Inc., Chicago, IL, USA) with a level of significance of α = 0.05.

## 3. Results

Descriptive characteristics and dimensions of breakfast intake of participants are shown in Table 1. Overall, it was observed that 7–21% and 4–11% of girls and boys, respectively, used to skip breakfast depending on the criteria used to define skipping breakfast. Although there were nonsignificant differences neither according to sex nor to study center in skipping breakfast, girls tended to skip breakfast more than boys (all *p* < 0.05). Finally, boys had higher levels of total daily physical activity and MVPA than girls (*p* < 0.05), while there were no significant differences according to the study center. 

BQI score was similar in boys and girls (Table 1**)**, whereas children from the ActiveBrains trial had significantly higher BQI score compared to children from the EFIGRO project, and therefore, had a better breakfast quality (*p* < 0.001). In contrast, BEDs and BEDb were similar in boys and girls, as well as in the two study centers (Table 1).

A higher percentage of boys met the criteria of having a dairy product included in the breakfast than girls (*p* < 0.02, Supplemental Table S2). The rest of the breakfast components included in the BQI were similar according to sex. Moreover, significantly higher number of participants from the ActiveBrains project met the criteria for BQI components such as cereals (*p* < 0.001), food rich in simple sugars (*p* < 0.05), MUFA-rich fats (*p* < 0.001), and MUFA/SFA ratio (*p* < 0.05), compared to children from the EFIGRO project (Supplemental Table S2). 

The associations of BQI score with cardiometabolic risk factors are shown in Table 2**.** BQI score was significantly and inversely associated with serum uric acid after adjusting for sex, age, center, maternal educational level, daily energy intake, and FMI (*p* < 0.05, Model 2, Table 2). However, this association was attenuated after further controlling for total physical activity (*p* < 0.07, Model 3, Table 2). Although there was a trend to significance between BQI score and GGT, BQI score was not significantly associated with the rest of cardiometabolic risk factors in children who were overweight or obese.

The associations between breakfast energy density and cardiometabolic risk factors are shown in Table 3. BEDs was significantly associated with total cholesterol regardless of potential confounders (*p* < 0.05, Model 2, Table 3) and of total physical activity (*p* < 0.05, Model 3, Table 3), as well as with HDL cholesterol regardless of potential confounders (*p* < 0.05, Model 2). Similarly, BEDs was positively associated with systolic blood pressure (*p* < 0.05, Model 2, Table 3). BEDb was significantly associated with HOMA after controlling for potential confounders including physical activity (*p* < 0.03 for all, Model 1, 2, and 3, Table 3). Furthermore, after categorizing children as being active (MVPA ≥ 60 min/day, *N* = 54) or inactive (MVPA < 60 min/day, *N* = 88), the current positive association between BEDb and HOMA remained statistically significant (β = 0.288; adjusted *p* = 0.025, and β = 0.250; adjusted *p* = 0.022 for active and inactive children, respectively). Finally, all the analyses examining the associations of BQI and energy density of breakfast were repeated including puberty stage instead of age into the models and the results did not substantially change (data not shown).

## 4. Discussion

The current study examined the association of several breakfast dimensions on cardiometabolic risk in children who were overweight or obese aged 8–12 years old. The main findings were that both breakfast quality and breakfast energy density were associated with some of the cardiometabolic risk factors studied. The BQI score was inversely associated with serum uric acid, while breakfast energy density from solids was related to higher total and HDL cholesterol and higher breakfast energy density from beverages was associated with higher levels of insulin resistance independently of physical activity levels in children with overweight/obesity. To the best of our knowledge, this is the first study examining the association of breakfast quality and energy density on cardiometabolic risk in children with overweight/obesity. 

In regards to skipping breakfast, a higher prevalence of skippers was reported by following the KIDMED criteria than by using the 24 h-recall questionnaire since the 24 h-recall criteria could only reflect two of the interviewed days and there might be children who were usual skippers, but consumed breakfast in the interviewed days by chance. Previous studies reported a breakfast skipping prevalence of 10–30% in children and adolescents from the United States and Europe [40], with an even higher prevalence in individuals with obesity [41]. 

Taking into account that two items from the BQI were related to monounsaturated fats, children from the ActiveBrains project could have higher BQI score because in the south of Spain the consumption of virgin olive oil is higher than in other regions. Indeed, children from the ActiveBrains study (south of Spain) tended to consume higher intakes of virgin olive oil in their breakfast whereas children from the EFIGRO study (north Spain) were more likely to consume less unhealthy alternatives such as biscuits, breakfast cereals with sugar added, or pastries and therefore, did not obtain a positive value in those items. 

We found a negative association between BQI score and serum uric acid levels albeit no with other cardiometabolic risk markers such as lipid, glycemic profile, or metabolic syndrome. Previous research showed that serum uric acid is related to cardiovascular disease [42,43], which in turn is an independent predictor for future cardiovascular mortality [44]. Milk proteins seem to decrease serum uric acid concentration in adults [45] and as BQI scored positively with the presence of dairy products in the meal, they could be one of the responsible components. A cross-sectional analysis of two Caucasian adult cohorts reported lower serum uric acid levels in participants with higher consumption of carbohydrates, calcium, vitamin B2, and lower fat intake [46]. The same study also examined individual food items, and high consumption of dairy products, fiber-rich bread, cereals, and fruits were negatively associated with serum uric acid. Of note, is that all these mentioned foods are components of BQI which score positively and therefore could partially explain our findings. However, it should be pointed that BQI was not associated with other cardiometabolic risk markers and metabolic syndrome. Overall, little is known about the influence of breakfast quality on cardiometabolic risk in children. Most studies in pediatric population examined just which were the health consequences of the consumption or skipping breakfast, whereas the few studies examining the quality of breakfast were carried out in adults [47], which hamper comparisons. Hence, as far as we are aware, there are no previous studies exploring the effect of breakfast quality in cardiometabolic health of children with overweight/obesity. 

Regarding energy density, we also observed that BEDs and BEDb were positively associated with both total and HDL cholesterol as well as with systolic blood pressure. The association of energy density from solids with HDL could be explained by the fact that virgin olive oil that was added to the toasts in breakfast was considered as solid as a whole (toast with virgin olive oil), since virgin olive oil is not drinkable. Cumulative evidence suggests that virgin olive oil may have a preventive role in coronary disease due to its monounsaturated fatty acids and polyphenolic compounds content [48,49]. In addition, considering that olive oil is energetically dense, the consumption of olive oil could be the responsible for the positive association between breakfast energy density from solids and HDL cholesterol. Furthermore, ultra-processed foods are energy-dense due to high salt, fat, and sugar content. The positive association we found between BEDs and systolic blood pressure could be explained by the high consumption of salt and sugars present in ultra-processed food consumed at breakfast, since both sugar and salt intakes have been associated with increased blood pressure [50,51]. 

One important finding of the current study is that BEDb might be associated with insulin resistance in children with overweight/obesity. Interestingly, this association was found regardless of being an active (meeting PA recommendations) or inactive (not meeting PA recommendations) children. A possible explanation for this relationship could be that almost all the kids of our projects tend to add soluble cocoa or similar sweeteners to their milk cups, which in turn, increase the energy density from these beverages. Moreover, some of the participants consumed artificial juices as a substitute of fruit or natural fruit juice. This fact would be in line with a study that reported that added sugars from liquid sources is associated with impaired glucose homeostasis and insulin resistance among youth at risk of obesity [52]. Similarly, Donin et al. reported a positive association between dietary energy density and insulin resistance and fat mass index in children aged 9–10 years [53], even though these associations were attenuated and became nonsignificant after adjustment for energy intake. According to another study in adults, total daily energy dietary energy density was associated with obesity and metabolic syndrome [54]. Taken into consideration, the fact that this positive association was observed in both active and inactive children, our hypothesis about the attenuation of the detrimental effects of the unhealthy breakfast by physical activity in cardiometabolic health was not confirmed. 

The current proposal for examining BQI includes an item about energy intake, but it does not include energy density of breakfast. However, taking into account our results on the association of energy density with insulin resistance, we believe that this dimension of breakfast should be also included when analyzing breakfast quality in youth. 

**Limitations and strengths**

The current study has several limitations. The cross-sectional design of the study should be considered as a study limitation. Dietary assessment was carried out by different researches which could bias the measurements. Nevertheless, in order to decrease the interpersonal variability, the same criteria were followed after reaching consensus. One of the strengths of the study is the homogeneity of our sample composed exclusively by children who were overweight or obese. Moreover, different dimensions of breakfast have been examined in order to have a more comprehensive study of breakfast adequacy. 

## 5. Conclusions

In conclusion, the findings of the current study suggest that both breakfast quality and breakfast energy density might be associated with cardiometabolic risk factors such as serum uric acid, cholesterol, and insulin resistance in children who are overweight obese. Moreover, energy density from beverages was associated with insulin resistance, regardless, a set of potential confounders were discovered both in both children meeting and not meeting physical activity recommendations. Results of the current study suggest that breakfast energy density should be considered as an additional dimension of breakfast intake. Therefore, nutritional education programs targeting the prevention of cardiometabolic disease in children who are overweight or obese should include strategies focused on promoting high quality and low energy density foods for breakfast.

## Figures and Tables

**Table 1:** Descriptive characteristics and dimensions of breakfast intake of participants.

	*N*	All	*N*	Girls	*N*	Boys	*N*	EFIGRO	*N*	ActiveBrains
**Biological characteristics**										
**Age (years)**	203	10.3 (1.1) ^§^	96	10.3 (1)	107	10.4 (1.2)	112	10.6 (1.1)	91	10 (1.1)
**BMI (kg/m^2^)**	203	26.1 (3.5)	96	25.9 (3.6)	107	26.3 (3.5)	112	25.5 (3.1)	91	26.9 (3.8)
**Obese (*N*, %)**	203	135 (65.5)	96	59 (61.5)	107	76 (71.0)	112	66 (58.9)	91	69 (78.8)
**High maternal educational level (*N*, %)**	203	54 (26.2)	96	25 (26)	107	29 (27.1)	112	28 (25)	91	26 (28.6)
**High puberty stage (*N*, %)** **^ϕ^**	197	45 (23.1)	88	35 (38.9)	98	10 (9.7)	102	32 (29.4)	84	13 (15.1)
**Waist circumference (cm)**	203	84.1 (10.5)	96	81.8 (10.4)	107	86.3 (10.2)	112	79 (7.6)	91	90.4 (10.3)
**FMI (kg/m^2^)**	184	10.7 (2.5)	86	10.7 (2.6)	98	10.6 (2.5)	110	10.1 (2.3)	74	11.5 (2.7)
**Abdominal fat (kg)**	183	1.7 (0.6)	86	1.7 (0.6)	96	1.6 (0.6)	110	1.5 (0.6)	73	1.8 (0.6)
**Systolic blood pressure (mmHg)**	197	105.2 (15)	94	103.9 (15)	103	106.4 (14.8)	112	96.2 (10.1)	85	117 (11.6)
**Diastolic blood pressure (mmHg)**	197	65.4 (9.8)	94	64.6 (9.5)	103	66.1 (10)	112	61.6 (8.3)	85	70.3 (9.5)
**Uric acid (mg/dL)**	188	4.7 (0.9)	87	4.9 (0.9)	101	4.6 (0.8)	108	4.7 (0.8)	80	4.7 (0.9)
**Cholesterol (mg/dL)**	197	170.3 (30.6)	93	170.4 (32.1)	104	170.2 (29.3)	110	171.7 (28.4)	87	168.6 (33.2)
**HDL-c (mg/dL)**	197	51.1 (11.6)	93	49 (11.1)	104	53 (11.8)	110	51 (11.2)	87	51.3 (12.1)
**LDL-c (mg/dL)**	189	102.8 (24.9)	88	104.2 (24.8)	101	101.5 (25)	110	104 (23.8)	79	101 (26.4)
**Triglycerides (mg/dL)**	197	89.9 (50)	93	96.6 (53.3)	104	84 (46,3)	110	83.7 (39.5)	87	97.8 (60.1)
**Glucose (mg/dL)**	197	85.9 (6.2)	93	84.8 (6.5)	104	86.9 (5.8)	109	85.4 (5.5)	88	86.5 (7.1)
**Insulin (µU/L)**	194	12.9 (8.5)	93	14.4 (10.7)	101	11.5 (5.6)	110	12.2 (5)	84	13.9 (11.6)
**HOMA**	192	2.8 (2.1)	92	2.9 (1.7)	100	2.6 (1.3)	109	2.6 (1.1)	83	3.2 (2.1)
**GGT (U/L)**	184	16.9 (5.9)	85	16.9 (6.8)	99	16.9(5.1)	108	16.3 (4.8)	76	17.7 (7.3)
**MetS (*N*, %)**	181	12 (6.1)	86	7 (7.8)	95	5 (4.9)	105	4 (3.7)	76	8 (9.3)
**MetS *z* score**	181	2.4 (3.7)	86	2.2 (3.9)	95	2.5 (3.7)	105	0.5 (2.9)	76	4.9 (3.3)
**Physical activity ^¥^**										
**Total physical activity (ENMO min/day)**	191	63 (15.4)	92	59.7 (13.7)	99	66.1 (16.5)	104	64.1 (16.4)	87	61.8 (14.3)
**MVPA (min/day)**	191	54.4 (20.9)	92	48.4 (18.3)	99	59.9 (21.7)	104	56.8 (21.7)	87	51.5 (19.6)
**Skipping breakfast (*N*, %)**										
***24 h-recall criteria***	203	11 (5.3)	96	7 (7.2)	107	4 (3.7)	112	4 (3.5)	91	7 (7.5)
***KIDMED item criteria***	172	26 (12.6)	80	17 (21.3)	91	9 (11)	96	14 (14.6)	75	12 (16)
**Breakfast quality**										
**BQI score (0–10)**	191	4.2 (1.3)	90	4.1 (1.3)	100	4.2 (1.2)	109	3.8 (1)	82	4.6 (1.5)
**Breakfast energy density**										
**From solids (BEDs)**	179	3.3 (1.1)	86	3.3 (1.2)	92	3.3 (1.1)	109	3.3 (1.3)	70	3.4 (0.9)
**From beverages (BEDb)**	191	0.5 (0.3)	88	0.5 (0.2)	102	0.5 (0.1)	109	0.5 (0.2)	82	0.5 (0.1)

BMI, Body mass index, FMI, fat mass index; HDL-c, High density lipoprotein cholesterol, LDL-c, low density lipoprotein cholesterol, HOMA, homeostasis model assessment for insulin resistance; GGT, gamma glutamyl transferase; MetS, metabolic syndrome; MVPA, moderate-to vigorous physical activity; BEDs, breakfast energy density from solids; BEDb, breakfast energy density from beverages.^§^ Values are means and standard deviations. ^Φ^ High puberty stage was considered as Tanner stage equal or above III. ^¥^ Physical activity was obtained from raw data based on Hildebrand et al. [30].

**Table 2:** Associations of Breakfast Quality Index (BQI) score with cardiometabolic risk factors.

	Model 1		Model 2		Model 3	
	β	*p*	β	*p*	β	*p*
**FMI *z*-score**	0.057	0.455	-	-	-	-
**Abdominal fat *z*-score**	0.024	0.752	−0.028	0.478	-0.047	0.235
**SBP (mmHg)**	0.047	0.384	0.042	0.426	0.034	0.533
**DBP (mmHg)**	0.034	0.625	0.029	0.674	0.041	0.553
**Uric acid (mg/dL)**	−0.128	0.091	−0.172	**0.028**	-0.151	0.060
**Cholesterol (mg/dL)**	−0.038	0.625	−0.036	0.659	-0.017	0.845
**HDL-c (mg/dL)**	−0.022	0.779	0.017	0.831	0.009	0.913
**LDL-c (mg/dL)**	−0.039	0.629	−0.046	0.582	-0.017	0.849
**Triglycerides (mg/dL)**	−0.008	0.913	−0.030	0.699	-0.020	0.804
**HOMA**	−0.016	0.845	0.022	0.779	0.020	0.809
**GGT (U/L)**	−0.136	0.096	−0.144	0.082	-0.161	0.061
**MetS *z*-score**	−0.018	0.779	−0.022	0.725	-0.011	0.869

FMI, Fat mass index; SBP, systolic blood pressure; DBP, diastolic blood pressure, HDL-c, High density lipoprotein cholesterol, LDL-c, low density lipoprotein cholesterol; HOMA, Homeostasis model assessment for insulin resistance; GGT, gamma glutamyl transferase; MetS, metabolic syndrome. Model 1 was adjusted for sex, age, center, maternal educational level, and energy intake; Model 2 was additionally adjusted for fat mass index; and Model 3 was further adjusted for total physical activity.

**Table 3:** Associations of breakfast energy density from solids (BEDs) and beverages (BEDb) with cardiometabolic risk factors.

			BEDs						BEDb			
	Model 1		Model 2		Model 3		Model 1		Model 2		Model 3	
	β	*p*	β	*p*	β	*p*	β	*p*	β	*p*	β	*p*
**FMI *z*-score**	−0.052	0.494	-	-	-	-	0.043	0.561	-	-	-	-
**Abdominal fat *z*-score**	−0.024	0.752	0.020	0.619	0.018	0.653	0.031	0.680	−0.007	0.848	−0.007	0.855
**SBP (mmHg)**	0.074	0.166	0.107	**0.045**	0.082	0.130	0.090	0.091	0.071	0.165	0.076	0.154
**DBP (mmHg)**	−0.031	0.654	0.018	0.794	−0.010	0.889	0.024	0.732	−0.007	0.922	−0.006	0.928
**Uric acid (mg/dL)**	−0.026	0.735	0.060	0.451	0.058	0.473	0.017	0.825	0.007	0.925	0.003	0.970
**Total cholesterol (mg/dL)**	0.144	0.061	0.165	**0.042**	0.181	**0.029**	0.032	0.681	0.021	0.798	0.018	0.824
**HDL-c (mg/dL)**	0.198	**0.008**	0.186	**0.016**	0.172	**0.032**	0.059	0.433	0.061	0.433	0.071	0.371
**LDL-c (mg/dL)**	0.094	0.231	0.109	0.189	0.127	0.136	0.040	0.610	0.035	0.674	0.029	0.731
**Triglycerides (mg/dL)**	0.001	0.985	0.030	0.698	0.039	0.625	−0.008	0.916	−0.027	0.731	−0.032	0.683
**HOMA**	0.050	0.549	0.076	0.354	0.107	0.205	0.203	**0.016**	0.190	**0.019**	0.190	**0.022**
**GGT (U/L)**	0.112	0.160	0.116	0.156	0.126	0.131	−0.008	0.919	−0.033	0.689	−0.030	0.714
**MetS *z*-score**	−0.035	0.586	0.012	0.846	0.003	0.958	0.051	0.425	0.023	0.710	0.020	0.744

BEDs, Breakfast energy density from solids; BEDb, breakfast energy density from beverages; FMI, Fat mass index; SBP, systolic blood pressure; DBP, diastolic blood pressure, HDL-c, High density lipoprotein cholesterol, LDL-c, low density lipoprotein cholesterol; HOMA, Homeostasis model assessment for insulin resistance; GGT, gamma glutamyl transferase; MetS, metabolic syndrome. Model 1 was adjusted for sex, age, center, maternal educational level, and energy intake; Model 2 was additionally adjusted for fat mass index; and Model 3 was further adjusted for total physical activity.

## Supplementary Materials

The following are available online at http://www.mdpi.com/2072-6643/10/8/1066/s1, Table S1: Components and criteria for the calculation of Breakfast Quality Index (BQI) score (14), Table S2: Breakfast Quality Index (BQI) components and number of children meeting the criteria (*N*, %).
