# Supplementary material for: Association of Breakfast Quality and Energy Density with Cardiometabolic Risk Factors in Overweight/Obese Children: Role of Physical Activity
Source: Nutrients. 2018 Aug 10;10(8):1066. doi: 10.3390/nu10081066 (PMC6116118; doi:10.3390/nu10081066)
Supplement: Supplementary file 1 [file nutrients-10-01066-s001.pdf]

Table S1. Components and criteria for the calculation of Breakfast Quality Index (BQI) score (14).

		Yes		No
Cereals and derivate	**+1**	Bread, non-sugar rich breakfast cereals	**0**	Biscuits, pastries, sugar rich breakfast cereals [ϕ]
Fruits and vegetables	**+1**	Fresh fruit, natural fruit juices, tomato	**0**	Artificial juices, jam [ϕ]
Dairy products	**+1**	Whole or skimmed milk, yoghurt, cheese	**0**	Dairy desserts [ϕ £]
Food rich in simple sugars	**+1**	<5% of total daily energy from simple sugars	**0**	≥5% of total daily energy from simple sugars
MUFA-rich fats	**+1**	Olive oil added by the consumer	**0**	Olive oil from biscuits or other fats such as butter [ϕ]
MUFA/SFA ratio	**+1**	≥ 2/1	**0**	< 2
Energy intake	**+1**	20-25% of daily energy intake from breakfast	**0**	<20% or >25% of daily energy intake from breakfast
Fruits, cereals and dairy product	**+1**	To include the 3 of the components	**0**	Not to be composed of three of the components
Calcium	**+1**	≥ 200mg	**0**	<200mg
Absence of butter or margarine	**+1**	Not to include butter or margarine in the breakfast	**0**	To include butter or margarine in the breakfast

MUFA, monounsaturated fatty acids; SFA, saturated fatty acids

[ϕ=] Not to consume the foods of these item also is punctuated as 0

£=Commercial chocolate milk-shakes, smoothies and rice-puddings, mousse, ice-creams or vegetal drinks

Sugared or flavored yoghurt, commercial chocolate milk-shakes and smoothies, rice-puddings, mousse, ice-creams, vegetal drinks.

Table S2. Breakfast Quality Index (BQI) components and number of children meeting the criteria (N, %).

	N	Girls	N	Boys	p	N	EFIGRO	N	ActiveBrains	p
**Cereals and derivate**	90	20 (22.2) [¥]	100	21 (21)	0.805	109	12 (11)	82	30 (36.6)	**<0.001**
**Fruits and vegetables**	90	7 (7.8)	100	12 (12)	0.591	109	11 (10.1)	82	8 (9.8)	0.360
**Dairy products**	90	73 (81.1)	100	89 (89)	**0.016**	109	94 (86.2)	82	69 (84.1)	0.885
**Food rich in simple sugars**	90	32 (35.6)	100	41 (41)	0.740	109	29 (26.6)	82	44 (53.7)	**0.001**
**MUFA-rich fats**	90	5 (5.6)	100	7 (7)	0.354	109	1 (0.9)	82	12 (14.6)	**<0.001**
**MUFA/SFA ratio**	90	1 (1.1)	100	4 (4)	0.410	109	1 (0.9)	82	4 (4.9)	**0.044**
**Energy intake**	90	9 (10)	100	5 (5)	0.405	109	7 (6.4)	82	7 (8.5)	0.634
**Fruits, cereals and dairy product**	90	5 (5.6)	100	3 (3)	0.587	109	2 (1.8)	82	6 (7.3)	0.172
**Calcium**	90	67 (74.4)	100	81(81)	0.504	109	81 (74.3)	82	68 (82.9)	0.355
**Absence of butter or margarine**	90	66 (73.3)	100	80 (80)	0.525	109	86 (78.9)	82	61 (74.4)	0.514

[¥] Values are the number and percentage of children who meet the criteria in both 24h-recalls.
